# Exploring the Roles of the *Swi2*/*Snf2* Gene Family in Maize Abiotic Stress Responses

**DOI:** 10.3390/ijms25179686

**Published:** 2024-09-07

**Authors:** Jiarui Han, Qi Wang, Buxuan Qian, Qing Liu, Ziyu Wang, Yang Liu, Ziqi Chen, Weilin Wu, Chuang Zhang, Yuejia Yin

**Affiliations:** 1 Institute of Agricultural Biotechnology/Jilin Provincial Key Laboratory of Agricultural Biotechnology, Jilin Academy of Agricultural Sciences (Northeast Innovation Center of Agricultural Science and Technology in China), Changchun 130033, China; 2 College of Agriculture, Yanbian University, Yanji 133002, China; 3 College of Agronomy, Jilin Agricultural University, Changchun 130118, China

**Keywords:** maize (*Zea mays* L.), *Snf2* gene family, phylogenetic analysis, stress response, qRT-PCR

## Abstract

The maize *Snf2* gene family plays a crucial role in chromatin remodeling and response to environmental stresses. In this study, we identified and analyzed 35 members of the maize *Snf2* gene family (*ZmCHR1* to *ZmCHR35*) using the Ensembl Plants database. Each protein contained conserved SNF2-N and Helicase-C domains. Phylogenetic analysis revealed six groups among the Snf2 proteins, with an uneven distribution across subfamilies. Physicochemical analysis indicated that the Snf2 proteins are hydrophilic, with varied amino acid lengths, isoelectric points, and molecular weights, and are predominantly localized in the nucleus. Chromosomal mapping showed that these genes are distributed across all ten maize chromosomes. Gene structure analysis revealed diverse exon–intron arrangements, while motif analysis identified 20 conserved motifs. Collinearity analysis highlighted gene duplication events, suggesting purifying selection. Cis-regulatory element analysis suggested involvement in abiotic and biotic stress responses. Expression analysis indicated tissue-specific expression patterns and differential expression under various stress conditions. Specifically, qRT-PCR validation under drought stress showed that certain *Snf2* genes were upregulated at 12 h and downregulated at 24 h, revealing potential roles in drought tolerance. These findings provide a foundation for further exploration of the functional roles of the maize *Snf2* gene family in development and stress responses.

## 1. Introduction

Maize is a cornerstone of global food security and economic development, and it ranks as one of the three major crops in Asia. The variability of the global environment poses significant threats to maize production worldwide, with various stresses including biotic and abiotic challenges [1,2]. Current research confirms that abiotic stresses such as extreme temperatures, high salinity, and drought are among the primary contributors to global maize yield losses [3]. It is estimated that a temperature increase of just one degree Celsius could lead to a 7.4% reduction in global maize production [4]. Consequently, elucidating the molecular mechanisms by which maize responds to abiotic stress is imperative.

In this context, transcription factors have garnered extensive attention in the scientific community. A central question in analyzing eukaryotic transcription factors is how these DNA-binding proteins interact with the chromatin template [5]. Research has shown that Swi2/Snf2 and related proteins can disrupt nucleosome stability, utilizing ATP to alter chromatin structure and nucleosome positioning, thereby facilitating the binding of transcription factors to chromatin. This is crucial for transcription regulation, DNA replication, and DNA damage repair [6,7,8]. Genetic studies of transcription regulation in Saccharomyces cerevisiae led to the discovery of various Swi (yeast mating type switching) and Snf (sucrose non-fermenting) genes [9]. Notably, the *Swi2* gene in Swi and the *Snf2* gene in Snf were found to be the same; hence, the gene is referred to as *Swi2*/*Snf2*. Plant genomes contain numerous Snf2-family proteins, some of which have been demonstrated to act as key developmental regulators during different stages in model plants such as Arabidopsis and rice [10]. Despite their crucial roles, studies of these proteins in maize have been relatively sparse.

As autotrophic organisms, plants can rapidly adjust their gene expression under environmental stress to ensure adaptability, genomic integrity, and enhanced future stress tolerance [11,12]. Although DNA methylation, histone variants, and modifications play significant roles in plant stress responses, the role of Snf2 chromatin remodeling pathways remains relatively understudied. Recent research highlights the prominence of *Snf2* genes under various abiotic stresses across multiple plant species [13]. In Arabidopsis, the *Snf2* family member *SPLAYED* (*SYD*) regulates the expression of many genes responsive to drought, salt, and temperature stress by remodeling chromatin structure [14]; another member, *BRAHMA* (*BRM*), participates in the abscisic acid (ABA) signaling pathway to modulate plant responses to drought stress [15]. In rice, the *Snf2* family member *OsCHR4* enhances drought resistance by increasing the waxy content of the leaf surface cuticle [16]. Transgenic Arabidopsis overexpressing *AtCHR12*, an *Snf2* family member, exhibit halted primary shoot growth and slowed main stem growth under drought and high-temperature stress [17]. Similarly, the tomato *SlCHR1* gene, similar to Arabidopsis *CHR12*, regulates growth retardation under salt stress while enhancing plant salt tolerance by maintaining chromatin structure stability [13]. Furthermore, maize *ZmCHB101* mutants show heightened sensitivity to salt stress due to chromatin alterations at the promoter, leading to disrupted expression of stress-responsive genes [18]. Additionally, *Snf2* factors are involved in hormone pathways, including cytokinin, auxin, and gibberellin signaling [19]. Arabidopsis *BRM* and *PKL* regulate the distribution of auxin and cytokinin responses, respectively, through *PIN* and *ARR* genes [20]. Thus, Snf2 chromatin remodeling factors have emerged as critical regulators of stress responses and developmental processes in various plant species.

The Snf2 protein family is characterized by conserved SNF2_N and Helicase_C domains [21]. Based on similarities in helicase-like regions and functional variations, the *Snf2* gene family is well represented in plant genomes. Arabidopsis has 41 members [22], rice has 40 [23], soybean has 66 [7], tomato has 45 [24], and barley has 38 members [25]. However, systematic identification and expression analysis under abiotic stress conditions for these genes in maize are relatively scarce. This study aims to thoroughly investigate the expression patterns of the maize *Swi2*/*Snf2* gene family members under abiotic stress conditions, providing insights for the cloning and characterization of maize-related functional genes and molecular marker-assisted breeding.

## 2. Results

### 2.1. Identification of the Maize Snf2 Gene Family

**Figure 1:**
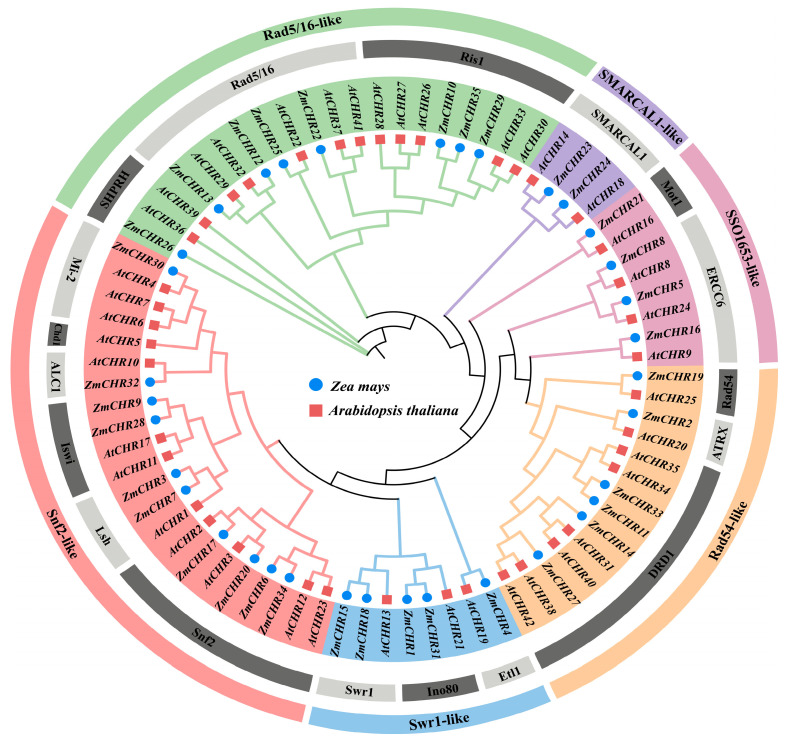
Phylogenetic tree of maize and Arabidopsis, in which *ZmCHR* genes are categorized into four main classes: Snf2-like, Swr1-like, Rad54-like, SSO1653-like, SMARCAL1-like, and Rad5/16-like. Within this phylogenetic tree, the *AtCHR* gene of Arabidopsis is specifically marked with a square and the *ZmCHR* gene of maize is specifically marked with a circle for easy identification.

Using the Ensembl Plants database, 35 members of the maize *Snf2* gene family were identified and systematically named *ZmCHR1* to *ZmCHR35* (Table S1), following the nomenclature currently used for Arabidopsis and rice and based on their physical chromosomal locations. All derived Snf2 proteins were found to contain the conserved SNF2-N domain and Helicase-C domain. To explore the evolutionary relationships between maize and Arabidopsis Snf2 proteins, phylogenetic analysis was conducted using the maximum likelihood method, incorporating 35 maize *Snf2* genes and 41 Arabidopsis *Snf2* genes. The resulting phylogenetic tree (Figure 1) shows that Snf2 proteins can be divided into six groups: Snf2-like, Swr1-like, Rad54-like, SSO1653-like, SMARCAL1-like, and Rad5/16-like, containing 18 different subgroups (Table S2). The distribution across these subfamilies was uneven, with the DRD1 evolutionary branch being the most expanded, comprising 10 members. It was followed by Rad5/16 with nine members, Ris1 with eight members, Snf2 also with eight members, and ERCC6 with six members. In contrast, other subfamilies such as SMARCAL1, Mi-2, and Iswi each had four members, while the Swr1, Ino80, SHPRH, and Lsh subfamilies each had three members. The subfamilies ALC1, Etl1, ATRX, Rad54, and Mot1 each contained only two *Snf2* members, and the Chd1 subfamily included just one Arabidopsis *Snf2* member. This systematic identification and classification provide a foundational framework for further studies on the functional roles of the maize *Snf2* gene family in development processes and stress responses.

### 2.2. Analysis of Physicochemical Properties of Maize Snf2-Family Proteins

The physicochemical properties of *ZmCHR*-family proteins were comprehensively analyzed using the ProtParam tool and the CELLO online platform. Although there are differences among members, these variations in physicochemical properties are relatively minor (Table 1). Analysis of protein structural characteristics revealed that the number of amino acids in these proteins ranged from 411 to 3828. The isoelectric point (pI) spanned a range from 5.07 (*ZmCHR20*) to 9.66 (*ZmCHR24*). The instability index varied between 40.88 and 64.6. The aliphatic index, which reflects the relative volume occupied by aliphatic side chains (alanine, valine, isoleucine, and leucine), ranged from 66.3 to 97.49, serving as an indicator of protein thermostability. Molecular weights were observed to be between 46.6 kDa and 415 kDa. Negative GRAVY values (from −0.853 to −0.153) indicated that all Snf2 proteins are hydrophilic. Subcellular localization predictions showed that most of the family members are predominantly localized in the nucleus, with only a few found in the cytoplasm and mitochondria. This analysis provides insights into the key structural features of maize *Snf2* genes and proteins. Furthermore, the goal of describing protein domain families is to better understand the functions of the proteins.

### 2.3. Chromosomal Distribution of Maize Snf2 Genes

**Figure 2:**
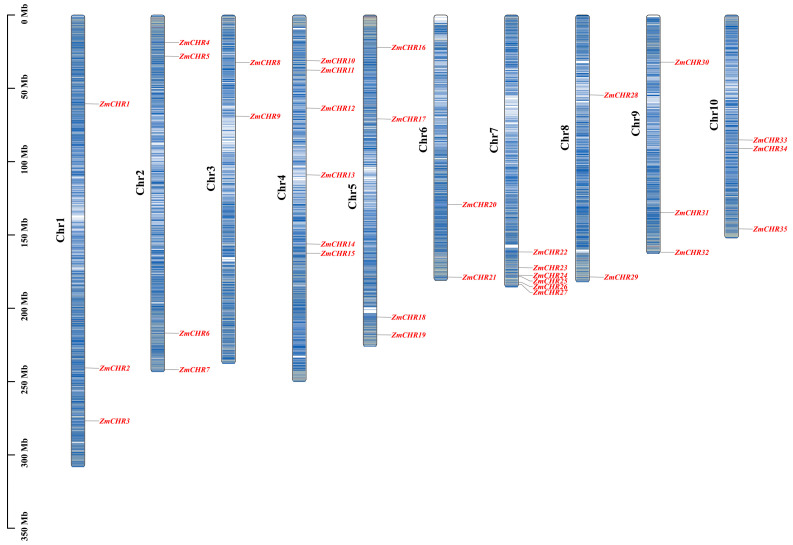
Chromosomal localization of the maize *ZmCHR* genes. Chromosome numbers are positioned on the left, with the scale on the left measured in megabases (Mb). An estimated genetic interval of 350 kb was set to accurately depict gene distribution. The color gradient from red to blue across the chromosomes represents gene density from high to low, where dark signifies areas of higher gene density and light indicates areas of lower gene density. Unmarked regions on the chromosomes represent genetic areas lacking gene distribution data.

The chromosomal distribution of the maize *Snf2* gene family was mapped across the maize genome, revealing that these genes are distributed on all ten pairs of chromosomes, albeit unevenly (Figure 2). Chromosomes 4 and 6 each host six *ZmCHR* genes, while chromosomes 2 and 5 each contain four genes. Chromosomes 1, 9, and 10 each have three genes, with the remaining chromosomes containing two genes each. This widespread chromosomal distribution underscores the evolutionary significance and potential functional diversity of the *ZmCHR* gene family in maize.

### 2.4. Gene Structure and Protein Domain Analysis of the Maize Snf2 Gene Family Members

**Figure 3:**
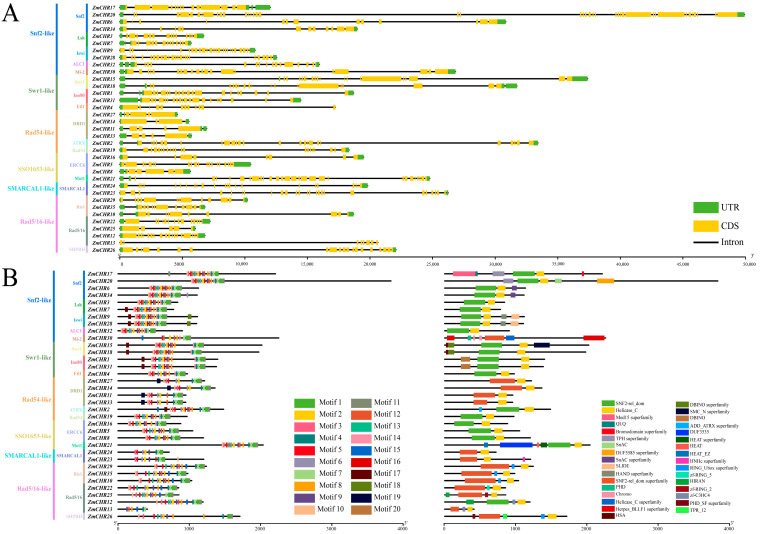
The structure, conserved domains, and motif patterns of maize *ZmCHR* genes. In part (**A**), the structure of *ZmCHR* genes is shown, where green boxes represent the untranslated regions (UTRs) at the 5′ and 3′ ends; yellow boxes indicate coding sequences (CDSs); and black lines denote introns, with numbers (0, 1, 2) indicating the phase of introns. Different conserved domains are marked with boxes of various colors. Part (**B**) depicts the motif patterns of *ZmCHR* members; Part C describes twenty conserved structural domains, with different colors corresponding to different structural domains. Additionally, the lengths of related gene structures and motif components can be estimated using the corresponding scale at the bottom of the panel.

Variations in exon–intron arrangement are a significant source of gene family diversity and contribute to plant diversity by causing changes in gene expression and function. Analysis of the exon/intron structures of the 35 *Snf2* family genes revealed considerable variation in exon counts, ranging from forty (*ZmCHR20*) to four (*ZmCHR14*) (Figure 3A). Further analysis indicated that members of the DRD1 subfamily generally possess fewer exons than members of other subfamilies. *Snf2*-family genes typically feature long sequences, with ten genes exceeding 20 kb and five genes exceeding 30 kb.

To study the homology among members of the maize *Snf2* gene family, 20 conserved motifs were identified (Figure 3B, Table S3). The number of motifs within each *ZmCHR* varied from six (*ZmCHR13*) to eighteen (*ZmCHR9* and *ZmCHR28*). Thirty members contained motifs 1 and 7, while thirty-four members, excluding *ZmCHR31*, contained motif 2, indicating that motif 2 is a relatively conserved pattern, likely involved in multiple cellular processes. Additionally, the composition of motifs is quite conservative within the same subfamily.

The goal of describing protein domain families is to better understand protein functions. To elucidate the roles of Snf2 family proteins, sequences were analyzed using Pfams and the Conserved Domain Database (CDD), identifying 20 conserved domains (Figure 3B). Analyses showed that nearly all members of the DRD1, SMARCAL1, SHPRH, Rad5/16, and Ris1 subfamilies, except *ZmCHR12*, possess the Helicase_C and SNF2-rel_dom superfamily domains. The Helicase_C and SNF2-rel_dom domains were found to be present in all subfamilies except Mi-2, DRD1, SMARCAL1, SHPRH, Rad5/16, and Ris1. The Chromo domain was exclusively found in the Mi-2 subfamily of maize *Snf2* genes.

### 2.5. Collinearity Analysis of the Maize Snf2 Gene Family

**Figure 4:**
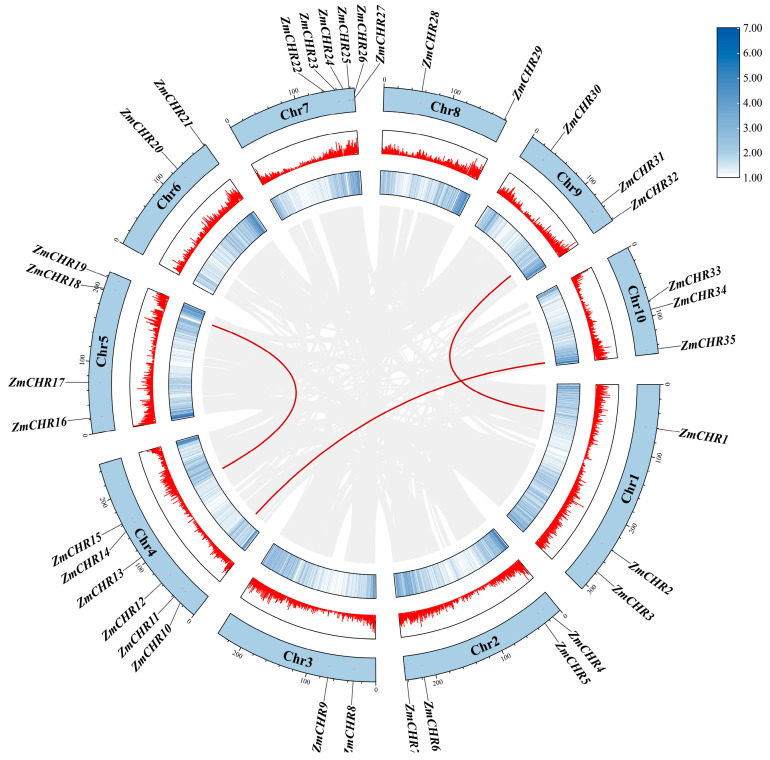
Collinearity among homologous maize *ZmCHR* genes. In the diagram, collinear blocks across the whole genome are depicted with a gray background, and red curves are used to connect pairs of duplicated *ZmCHR* genes.

To elucidate the collinearity among *Snf2* family genes, intraspecific analyses within maize and interspecific analyses among maize, Arabidopsis, rice, and Sorghum were conducted using MCScanX. This analysis, facilitated by TBtools, assessed the thirty-five identified *ZmCHR* genes and identified three pairs of duplicated genes: *ZmCHR1*-*ZmCHR31*, *ZmCHR35*-*ZmCHR10*, and *ZmCHR15*-*ZmCHR18* (Figure 4). Each pair of duplicated genes belongs to the same subgroup, suggesting that these chromosomal segments may have undergone duplication events without complete diversification, potentially leading to functional redundancy. The synonymous and nonsynonymous substitution rates for these duplicated gene pairs were calculated using the Simple Ka/Ks Calculator in TBtools. Results indicated that all six *ZmCHR* tandem duplicate sequences had a Ka/Ks ratio less than one (Table S4). It is widely accepted that a Ka/Ks ratio less than one indicates negative or purifying selection, a ratio of one indicates neutral selection, and a ratio greater than one suggests positive selection. These findings suggest that the duplicated genes in maize have undergone purifying selection aimed at eliminating deleterious mutations. This purifying selection underscores the evolutionary pressure to maintain the fundamental functions of the Snf2 gene family in maize and reduce redundancy, further revealing the genetic stability and functional integrity of these genes.

**Figure 5:**
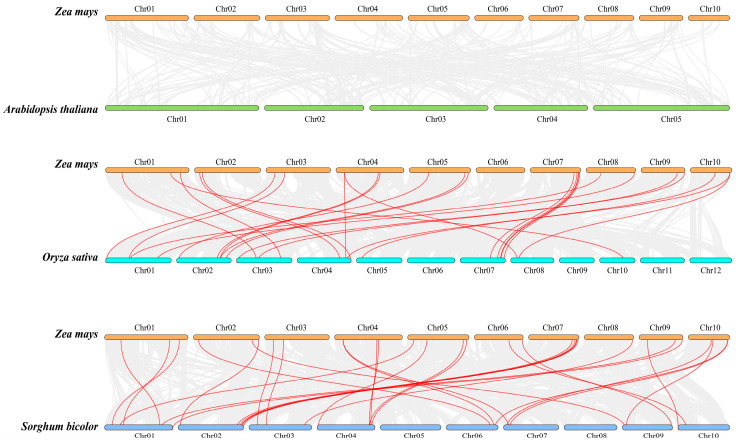
The collinearity diagram of *Snf2* genes between *Z. mays*, *A. thaliana*, *O. sativa*, and *S. bicolor*. The gray line in the background represents the collinear gene pairs between the two species, and the *Snf2* collinear genes are connected by red lines.

Additionally, the orthologous relationships of *Snf2* genes between *Zea mays* and two other Poaceae species (*Oryza sativa* and *Sorghum bicolor*) were investigated to explore the origins and evolutionary relationships of the *Snf2* genes (Figure 5). There are 31 orthologous gene pairs between *Z. mays* and *S. bicolor*, and 26 between *Z. mays* and *O*. *sativa*. No orthologous gene pairs were found with *Arabidopsis thaliana*, and the extensive homology between *Z. mays*, *O. sativa*, and *S. bicolor* supports their close evolutionary relationships within the Poaceae family.

### 2.6. Analysis of Cis-Regulatory Elements in the Promoters of Maize Snf2 Genes

**Figure 6:**
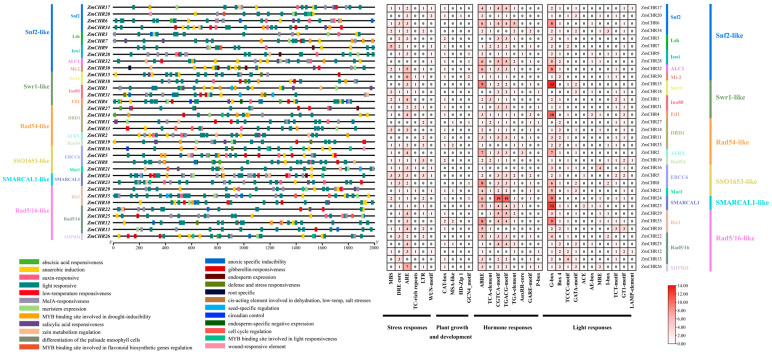
The *ZmCHR* gene family promoter sequences were used to predict cis-acting elements; the predicted cis-acting elements were organized and visualized.

To explore the potential regulatory functions of *Snf2* genes under abiotic and biotic stresses, the 2 Kb promoter sequences of *Snf2* genes were analyzed to detect cis-regulatory elements (CREs). Eight plant-hormone-responsive cis-regulatory elements were identified in the promoter regions of *Snf2* genes, including ABRE elements, TCA element, CGTCA motif, TGACG motif, TGA element, AuxRR-core, GARE-motif, and P-box (Figure 6). ABRE (abscisic acid-responsive) elements were identified in 32 *Snf2* genes, while CGTCA (MeJA-responsive) and TGACG (MeJA-responsive) motifs were found in 30 *Snf2* genes each. Additionally, 33, 31, 22, 16, 19, and 33 *ZmCHR* genes contained G-Box (abscisic acid- and light-responsive), ARE (antioxidant response element), DRE (dehydration-responsive element), LTR (low-temperature responsive), MBS (drought-inducible element), and CAT-box, respectively. The visualization of cis-regulatory elements in plant promoters was facilitated using TBtools, revealing that the cis-regulatory elements in 35 *ZmCHRs* are related to light response, abscisic acid response, methyl jasmonate response, anaerobic induction, drought induction via MYB-binding sites, and low-temperature response (Figure 6). These results suggest that the maize *Snf2* gene family may be involved in regulating responses to abiotic and biotic stresses, as well as hormone signaling pathways.

### 2.7. Expression Analysis of Maize Snf2 Genes in Various Tissues

**Figure 7:**
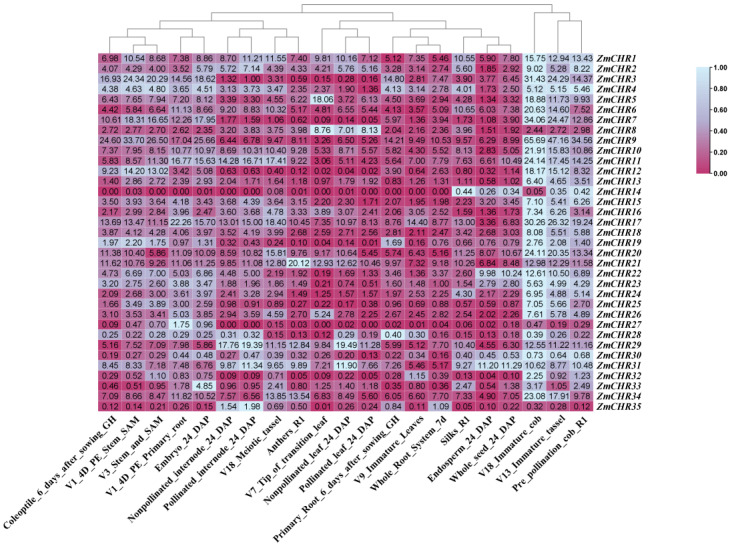
The transcriptomic expression levels of the *ZmCHR* gene family across various tissues, including the embryos, endosperm, immature tassel and cob, internodes and leaves before and after pollination, anthers, silks, complete root system, and whole seeds. Note: DAP, days after pollination; GH, greenhouse; R1, reproductive 1; SAM, shoot apical meristem; 7 d, 7 days after sowing.

In examining the expression patterns of *Snf2* genes across different tissues and developmental stages, we explored the dynamic changes within the maize *Snf2* gene family. *Snf2* genes exhibited tissue-specific expression profiles. Members of the maize *Snf2* family were predominantly expressed during the reproductive growth stages, specifically in immature tassels and cobs. Minimal to no expression was observed during early developmental stages (coleoptile 6 days after sowing GH and primary root 6 days after sowing GH), vegetative growth stages (stem apical meristem, young leaves, and early whole root system), grain development stages (endosperm and whole seed at 24 days), and other critical tissues (anthers, rachises before pollination, and leaves and internodes before and after pollination). The immature rachis represents a crucial stage in the formation of maize reproductive organs, characterized by rapid cell proliferation and differentiation. The *Snf2* gene family typically encodes core subunits of chromatin remodeling complexes that play key roles in regulating gene expression, DNA repair, and chromatin conformation changes. Therefore, the elevated expression of *Snf2* genes during these periods of high proliferation and differentiation is logical. Notably, *ZmCHR27* was highly expressed in early primary roots, *ZmCHR5* and *ZmCHR8* showed higher expression levels in mid-vegetative-growth leaves, *ZmCHR21* was highly expressed in anthers, and *ZmCHR35* exhibited increased expression in internodes post-pollination. These findings suggest their potential significance in plant growth and development. On the other hand, the high expression of *ZmCHR33* in embryos at 24 days suggests their involvement in seed germination (Figure 7, Table S5).

### 2.8. Expression Analysis of Maize Snf2 Genes under Various Stress Conditions

**Figure 8:**
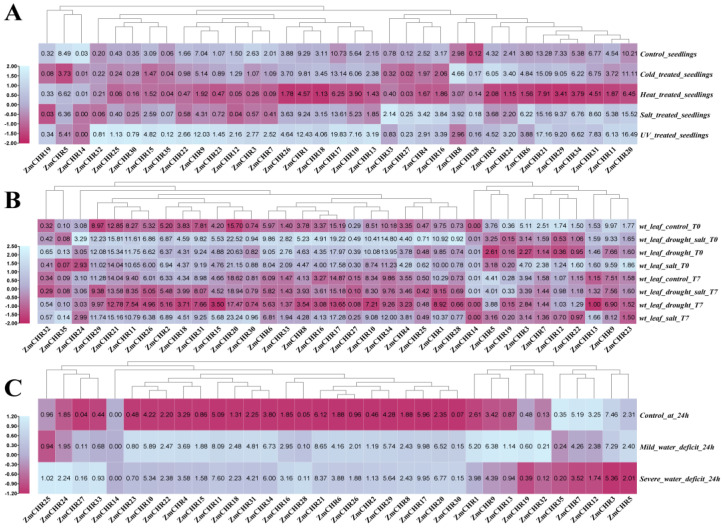
The expression levels of maize *ZmCHR* gene family members under abiotic stresses are depicted in (**A**). (**A**) depicts the expression levels of *ZmCHR* in the whole above-ground tissues of maize seedlings under cold, heat, salt, and ultraviolet stresses; (**B**) depicts the expression levels of *ZmCHR* in maize leaves under different drought and salt stresses; and (**C**) depicts the expression levels of *ZmCHR* in the primary roots of maize seedlings under different water deficit stresses. Note: for cold stress, seedlings were incubated at 5 °C for 16 h; for heat stress, seedlings were incubated at 50 °C for 4 h; for high-salt stress, plants were watered with 300 mM NaCl 20 h before tissue collection; for UV stress, the UV stressor was applied for 2 h using a UV-B lamp under growth chamber conditions before tissue collection; T0, after ten days of continuous treatment; T7, after ten days of treatment with normal watering for seven days to recover from stress; Mild_water_deficit indicates moderate water deficit treatment (−0.2 Mp water potential); Severe_water_deficit indicates severe water deficit treatment (−0.8 Mp water potential).

To investigate the expression dynamics of maize *Snf2* genes under abiotic stress conditions, RNA-seq data for the inbred line B73 were retrieved from the GDB database (https://maizegdb.org/ (accessed on 6 June 2024)). We analyzed the expression patterns of maize *Snf2* family members in aerial tissues of seedlings under cold, heat, high-salinity, and UV stress; in leaves under drought, salinity, and combined stress; and in primary roots of seedlings under varying degrees of drought stress, as documented by Makarevitch [26] et al. (2015), Forestan [27] et al. (2016), and Opitz [28] et al. (2014). Our findings indicated significant downregulation of *ZmCHR19*, *ZmCHR5*, *ZmCHR14*, *ZmCHR3*, *ZmCHR7*, and *ZmCHR16* under cold stress, while *ZmCHR8*, *ZmCHR28*, *ZmCHR2*, and *ZmCHR24* were notably upregulated. All studied genes were downregulated under high-temperature stress. Under salt stress, *ZmCHR19*, *ZmCHR5*, *ZmCHR14*, *ZmCHR3*, and *ZmCHR7* showed significant downregulation, whereas *ZmCHR33*, *ZmCHR27*, *ZmCHR4*, *ZmCHR16*, *ZmCHR28*, *ZmCHR6*, and *ZmCHR31* were markedly upregulated. Under UV stress, *ZmCHR5* and *ZmCHR14* were significantly downregulated, while other genes (except *ZmCHR19*, *ZmCHR33*, *ZmCHR8*, *ZmCHR2*, *ZmCHR6*) showed significant upregulation. T0 represents ten days after stress treatment, and T7 represents seven days of normal conditions following the ten-day treatment, marking recovery from stress. Ten days after combined stress, *ZmCHR24* to *ZmCHR28* showed marked upregulation, while *ZmCHR19* to *ZmCHR22* were notably downregulated. Under mild drought conditions, *ZmCHR23* to *ZmCHR32* were significantly upregulated, whereas *ZmCHR35*, *ZmCHR7*, and *ZmCHR12* were downregulated. Under severe drought, *ZmCHR25*, *ZmCHR24*, *ZmCHR27*, and *ZmCHR33* were markedly upregulated, and *ZmCHR35*, *ZmCHR7*, and *ZmCHR12* were significantly downregulated (Figure 8, Tables S6–S8).

### 2.9. Analysis of Maize Snf2 Gene Expression under Drought Stress and qPCR Validation

**Figure 9:**
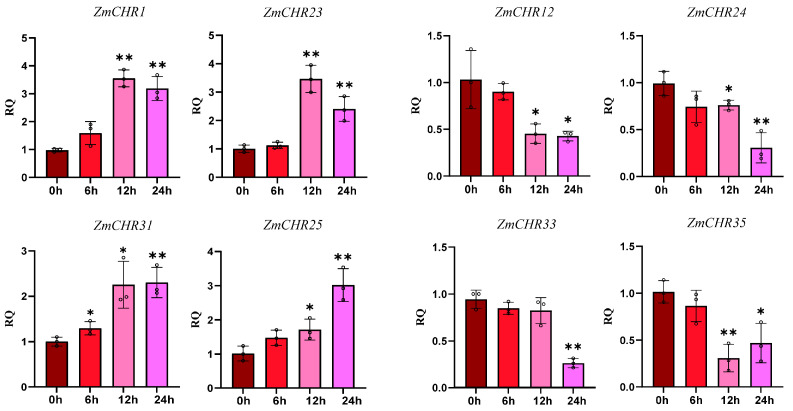
Quantitative RT-PCR analysis of selected representative genes in the maize *ZmCHR* family. Vertical bars indicated the standard deviations. The values represent the mean ± standard deviation (SD) of three independent replicates. An asterisk indicates that the corresponding gene is significantly upregulated or downregulated compared with the 0 h status (* *p* < 0.05, ** *p* < 0.01, *t*-test).

Based on the gene expression profiles of maize *Snf2* genes under drought stress, four genes were randomly selected among the genes upregulated and downregulated at 24 h, respectively, and analyzed by qRT-PCR at 0, 6, 12, and 24 h of drought stress. The results showed that *ZmCHR1* and *ZmCHR23* exhibited an upward trend at 12 h, followed by a decrease at 24 h, although they remained upregulated compared to the control group. *ZmCHR25* displayed a rapid increase in expression levels after 24 h of drought stress. *ZmCHR31* rose sharply at 12 h after stress and remained constant at 24 h. In contrast, *ZmCHR35* and *ZmCHR12* exhibited a downward trend at 12 h. *ZmCHR24* and *ZmCHR33* showed significant decreases starting at 24 h (Figure 9). The expression patterns of the selected genes were consistent with transcriptome data. Overall, certain *Snf2* genes in maize respond to drought stress, and the observed divergent trends may indicate varying degrees of drought tolerance in maize.

## 3. Discussion

### 3.1. Functional Domains and Evolutionary Dynamics of the Maize Snf2 Gene Family

Snf2 proteins, acting as core ‘motors’, have been characterized at the whole-genome level in various crops including Arabidopsis, wheat, rice, and sorghum. In this study, we systematically identified and characterized 35 *Snf2* family genes on 10 chromosomes in maize. In previous studies, 38 genes encoding Snf2 proteins were identified in barley and sorghum [25,29], 41 in Arabidopsis [22], 45 in tomato [24], and 40 in rice [23], and the number of genes encoding Snf2 proteins in maize was less than the numbers in the above crops, suggesting that the differences in species and genome sizes are not directly related to the number of *Snf2* family genes, which lays the foundation for revealing multiple chromatin remodeling mechanisms mediated by different Snf2 protein subfamilies. As catalytic ATPase subunits of chromatin remodeling complexes, Snf2 proteins contain conserved helicase-C and SNF2-N domains [30], enabling their involvement in multiple DNA processes, including replication [31], transcription [32], homologous recombination [33], and repair [34]. These conserved domains mediate the varied chromatin remodeling activities of Snf2 proteins in these fundamental nuclear events. Such functional diversification allows Snf2 proteins to participate in regulating various plant-specific developmental processes and stress response pathways [35].

Phylogenetic analysis of the maize Snf2 protein family classified the thirty-five maize *Snf2* members into six major groups and seventeen subfamilies, with each major group containing between two and ten maize members. Interestingly, most subfamilies (Snf2, Iswi, ALC1, EtI1, ATRX, Rad54, ERCC6, Mot1, and SMARCAL1) exhibit a 1:1 orthologous conservation pattern between maize and Arabidopsis, suggesting that these subfamilies are evolutionarily more conserved than others. This was similarly found in wheat, Arabidopsis, and rice [36].

### 3.2. Function and Diversity: The Role of the Maize Snf2 Gene Family in Chromatin Remodeling

The *Snf2* gene family in maize plays a crucial role as a component of the chromatin remodeling complex, which is essential for plant growth and environmental adaptability [37]. Due to the sedentary growth nature of plants, they must adapt in situ to external changes [38]. This unique mode of growth means that plants exhibit distinct epigenetic regulatory mechanisms compared to animals. Historically, there has been a relative lack of systematic research into the classification, subunit composition, and role of chromatin remodeling complexes in regulating transcription in plants. However, with the integration of technologies such as mass spectrometry, protein interaction network analysis, high-throughput sequencing, and protein structure resolution, significant progress has been made in this field [39]. The SWI/SNF complex, first identified through genetic screening in yeast, includes the Swi2/Snf2 protein, which contains evolutionarily conserved Helicase_C and SNF2_N domains. These domains alter chromatin structure through their catalytic ATPase activities, impacting DNA accessibility and associated biological processes. Additionally, maize Snf2 proteins include supplementary domains that demonstrate subfamily-dependent functional diversity. In contrast to all others, members of the DRD1, SMARCAL1, Ris1, Rad5/16 (except *ZmCHR12*), and SHPRH subfamilies contain a SNF2-related domain superfamily member upstream of the Helicase_C domain, whose ATPase activity regulates the chromatin remodeling process, thereby influencing the accessibility of DNA and its related biological processes.

### 3.3. Differential Expression Patterns of Maize Snf2 Gene in Various Organs

Spatio-temporal expression patterns of genes during reproductive development may provide clues to elucidate their potential functions. Most of the *Snf2* genes in maize are expressed during the reproductive growth stage and are particularly highly expressed in immature tassels and cobs. This suggests that *Snf2* family genes may activate cell division and differentiation. Yu et al. found that the core subunit of the SWI/SNF chromatin remodeling complex, *ZmCHB101*, is involved in vesicle division and development and that transgenic lines of the *ZmCHB101* RNA interference (RNAi) construct had impaired spike and rachis development [40]. The *Snf2* gene *ZmDDM1* has been reported in maize, and its homologs are abundantly expressed in the embryo; once the gene is deleted, abnormal cell proliferation occurs during the early stages of kernel development [41]. There is also evidence for this in other crops. Chen et al. found that the *Snf2* gene family has multiple regulatory roles in spike development in barley [25]. Several *Snf2* genes in rice have been reported to be specifically expressed in the early development of the pistil [42]. *OsCHR721* interacts with *OsRPA1a*, which is involved in DNA repair and plays a key role in male and female reproductive development [43]. Several previous studies in Arabidopsis have also shown that the Snf2 gene is essential for regulating flower developmental processes such as floral transition [44], floral organ characterization [45], and inflorescence structure [46]. These findings suggest that the *Snf2* gene has potential research implications in maize growth and development.

### 3.4. Role of the Maize Snf2 Gene Family in Response to Abiotic Stress

When plants are subjected to abiotic stresses, they undergo a variety of physiological and biochemical responses that alter the gene expression program to rapidly adapt to adverse conditions. To investigate the expression pattern of the *Snf2* gene under abiotic stress, we performed a computer-simulated cis-regulatory element analysis of the putative promoter region of the maize *Snf2* gene, and we identified several cis-regulatory elements associated with abiotic stresses such as gibberellic acid and drought. Subsequent analysis of the *Snf2* gene in different tissues of maize under different stresses using publicly available gene expression profiling databases revealed a significant upregulation/downregulation trend of the maize *Snf2* gene under high temperature, low temperature, salt, UV, and drought stresses. This gives a preliminary validation of the *Snf2* gene family in response to abiotic stresses. Finally, we conducted qRT-PCR experiments using eight genes selected in the expression profiles for further validation. The results showed that all eight selected genes responded to drought stress.

The response of the *Snf2* gene family to abiotic stresses has been reported in several plant species. In Arabidopsis, the *SWI*/*SNF* subunits are involved in many biotic and abiotic stress responses; the *PKL* mutant pkl is highly sensitive to cold stress, resulting in reduced cotyledon greening and reduced primary root elongation at high salinity [47]; and *BRM* negatively regulates drought tolerance in Arabidopsis [48]. Both *AtLFR* in Arabidopsis and its homologous gene *GmLFR1* in soybean are negative regulators of drought tolerance [49]. A previous report showed that overexpression of the SWI/SNF complex subunit *OsSWI3C* in rice resulted in reduced drought tolerance [50], and *OsCHR710* was upregulated under drought stress [42]. The involvement of the *SWI2*/*SNF2* genes in UV-B tolerance and acclimatization was mentioned in an analysis showing that chromatin remodeling proteins are responsive to UV-B [51]. These findings emphasize the close link between at least some of the *Snf2* genes and plant stress responses, as well as their possible function in drought tolerance, highlighting their key role in addressing related environmental challenges.

## 4. Materials and Methods

### 4.1. Identification and Physicochemical Property Prediction of the Maize Snf2 Gene

The maize genome and GFF annotation files were downloaded from the Ensembl Plants database (https://plants.ensembl.org/index.html, accessed on 1 June 2024). The amino acid sequence of the Arabidopsis *Snf2* gene was retrieved from The Arabidopsis Information Resource (TAIR) (https://www.arabidopsis.org/, accessed on 1 June 2024). Local BLAST searches were conducted using the ‘Blast Compare Two Seqs’ module within TBtools (v2.097), setting the E-value threshold to 10^−5^. Furthermore, characteristic structural domains of the maize *Snf2* gene family (PF00176 and PF00271) were identified using the Pfam database (http://pfam.xfam.org/, accessed on 4 June 2024) through a Simple HMM Search. The intersection of sequence IDs from two screening steps was taken to obtain the longest amino acid sequence variant within different transcripts using the ‘Fasta Get Representative’ module in TBtools (v2.097). Finally, sequences with incomplete conserved domains were excluded using the Conserved Domain Database (CDD) search tool provided by the National Center for Biotechnology Information (https://www.ncbi.nlm.nih.gov/Structure/bwrpsb/bwrpsb.cgi, accessed on 4 June 2024).

The physicochemical properties of the predicted maize Snf2 protein were calculated using the ProtParam tool (https://web.expasy.org/protparam/, accessed on 4 June 2024). These properties included amino acid sequence length, theoretical isoelectric point (pI), molecular weight (Mw), instability index, aliphatic index, and grand average of hydropathicity (GRAVY) index. Subcellular localization of the maize *Snf2* gene was predicted using the CELLO (v.2.5) online tool (http://cello.life.nctu.edu.tw/, accessed on 4 June 2024).

### 4.2. Chromosomal Localization of the Maize Snf2 Gene

Chromosomal position information for the maize *Snf2* gene was extracted from the maize genome annotation file. Visualization of this chromosomal localization was performed using the ‘Gene Location Visualize from GTF/GFF’ module in TBtools (v2.097).

### 4.3. Phylogenetic, Gene Structure, and Conserved Motif Analysis of the Maize Snf2 Gene

Amino acid sequences of the *Snf2* gene from maize and Arabidopsis were selected for phylogenetic analysis. Multiple sequence alignments were conducted using MUSCLE, followed by trimming with trimAL. A phylogenetic tree was constructed using the maximum likelihood (ML) method in the IQ-TREE software (1.6.12), with a bootstrap value set to 5000. The resulting phylogenetic tree was visualized and embellished using iTOL (https://itol.embl.de/, accessed on 5 June 2024).

The motif architecture of the maize *Snf2* gene family members was analyzed using the online MEME suite (http://www.OMIcsclass.com/article/67, accessed on 5 June 2024). The maximum number of motifs was set to 20, with minimum and maximum motif widths set at 6 and 50, respectively. The gene structure of maize *Snf2*, including coding sequences (CDSs) and untranslated regions (UTRs), was extracted from the maize genome annotation file. Visualization of these features was performed using TBtools (v2.097).

### 4.4. Collinearity Analysis and Gene Duplication of the Maize Snf2 Gene

Homologous *Snf2* genes within maize were identified using the BLASTP program, with an E-value threshold set to <10^−5^. Collinearity among maize *Snf2* genes was analyzed using the default parameters in the MCScanX program. Homologous blocks and duplicated *Snf2* gene pairs were visualized using TBtools. Genome sequences and annotation files for Arabidopsis and rice were downloaded from the Phytozome v13 website (https://phytozome-next.jgi.doe.gov/, accessed on 5 June 2024), and those for sorghum were obtained from the NCBI website (https://www.ncbi.nlm.nih.gov/, accessed on 5 June 2024). Collinearity between maize and Arabidopsis, rice, and sorghum was analyzed using the MCScanX program.

### 4.5. Analysis of Cis-Acting Elements in the Promoter Regions of the Maize Snf2 Gene

The 2000 bp region upstream of the start codon (ATG) of each maize *Snf2* gene was considered as the promoter sequence. These promoter sequences were extracted using TBtools (v2.097) and analyzed using the PlantCARE database (https://bioinformatics.psb.ugent.be/webtools/plantcare/html/, accessed on 6 June 2024) to predict cis-regulatory elements. Visualization and summary of cis-elements related to stress response, plant growth and development, plant hormone responsiveness, and light responsiveness were performed using the Basic Biosequence View and HeatMap modules in TBtools (v2.097).

### 4.6. Expression Analysis of the Snf2 Gene Family in Maize

Transcriptomic data for the maize *Snf2* gene in various tissues were analyzed using the qTeller tool (https://qTeller.MaizeGDB.org, accessed on 6 June 2024) MaizeGDB, which included tissues such as the immature tassel and rachis, embryo, endosperm, unpollinated internodes, unpollinated leaves, pollinated internodes, pollinated leaves, stem apical meristem, complete root system, and whole seed (https://qTeller.MaizeGDB.org, accessed on 6 June 2024). These analyses were conducted using RNA-Seq data from Stelpflug et al. [52]. Additionally, the transcriptional expression levels of the maize *Snf2* gene under various abiotic stress conditions were examined using RNA-Seq data from studies by Makarevitch [26], Forestan [27], and Opitz [28], among others.

### 4.7. Plant Material and Stress Treatment Procedures

Maize inbred line B73 was used as the plant material. Seeds were sterilized with 75% alcohol and 2% sodium hypochlorite before being planted in autoclaved nutrient soil. The plants were subsequently grown in a controlled greenhouse environment, maintained at 25 °C with a photoperiod of 8 h of light followed by 16 h of darkness. At the three-leaf stage, seedlings with uniform growth were selected for drought stress experiments. To simulate drought conditions, these seedlings were treated with 15% PEG6000. The second fully expanded leaves from three seedlings were collected at 0, 6, 12, and 24 h post-treatment and immediately frozen in liquid nitrogen at −80 °C for subsequent RNA extraction.

### 4.8. RNA Extraction and Quantitative Real-Time PCR Analysis

A 150–300 mg mass of leaves was collected at each stress time point, then added to liquid nitrogen and quickly ground into powder. The resulting powder was divided into triplicates, and then RNA was extracted using an RNA extraction kit (CW Bio, Taizhou, China). The quality of the extracted RNA was assessed through gel electrophoresis and quantified using a NanoDrop ND-2000 spectrophotometer (Thermo Scientific, Wilmington, DE, USA). RNA samples that met the quality standards were pooled and reverse-transcribed into cDNA, with concentrations subsequently measured. The cDNA from each sample was then uniformly diluted to 200 ng/μL for use as templates in real-time quantitative PCR (qRT-PCR) experiments.

To explore the expression patterns of maize *Snf2* genes under abiotic stress, eight *ZmCHR* genes were randomly selected for qRT-PCR analysis. Specific primers for these genes were designed using NCBI’s Primer-BLAST tool (https://blast.ncbi.nlm.nih.gov/Blast.cgi, accessed on 6 June 2024), and qRT-PCR was performed using SYBR™ Green PCR Master Mix (Thermo Fisher Scientific, Waltham, CA, USA). Each sample was analyzed in triplicate, with the 0 h stress time point serving as the control. The results were assessed using the relative quantification (RQ) method. The reaction system and conditions for RT-qPCR were based on the method outlined by Mo et al. [53]. All primer sequences utilized in this study are provided in Supplementary Table S9.

## 5. Conclusions

This study identified and comprehensively analyzed 35 *ZmCHR* genes in the maize genome. The structure and chromosomal locations of these genes were detailed, a phylogenetic tree was constructed, and the properties of their encoded proteins were analyzed. Cis-regulatory element and transcriptional expression analyses suggest that the SWI/SNF complex may play a significant role in maize’s response to environmental stress. This research provides a critical foundation for understanding the composition of the SWI/SNF complex in maize and paves the way for further investigation into its role in abiotic stress responses.

## Figures and Tables

**Table 1:** Characteristics of gene structures and protein properties of maize *ZmCHR* family members.

Gene Name	Sequence ID	Length (aa)	MW (kDa)	pI	Instability Index	Aliphatic Index	GRAVY	Predicted Location(s)
*ZmCHR1*	Zm00001eb017000_T001	1398	159.89	8.11	48.88	83.53	−0.529	Nuclear
*ZmCHR2*	Zm00001eb046650_T001	1481	168.18	5.46	51.9	76.91	−0.674	Nuclear
*ZmCHR3*	Zm00001eb055640_T002	837	94.16	5.46	45.07	92.19	−0.417	Nuclear
*ZmCHR4*	Zm00001eb073870_T001	973	109.04	6.12	52.05	81.01	−0.525	Nuclear
*ZmCHR5*	Zm00001eb076670_T001	1048	116.95	5.85	42.11	83.11	−0.533	Nuclear
*ZmCHR6*	Zm00001eb108690_T001	1129	128.88	6.78	52.49	80.88	−0.629	Nuclear
*ZmCHR7*	Zm00001eb117870_T001	779	88.40	6.21	44.7	90.8	−0.461	Cytoplasmic
*ZmCHR8*	Zm00001eb127010_T001	1198	133.40	7.64	48.2	79.47	−0.609	Nuclear
*ZmCHR9*	Zm00001eb131790_T002	1114	128.43	5.22	49.03	72.56	−0.851	Nuclear
*ZmCHR10*	Zm00001eb171780_T003	1033	114.16	7.75	47.19	80.92	−0.455	Nuclear
*ZmCHR11*	Zm00001eb173050_T002	952	106.75	6.61	40.88	86.13	−0.418	Nuclear
*ZmCHR12*	Zm00001eb176980_T001	1193	132.33	8.52	48.01	87.04	−0.248	Nuclear
*ZmCHR13*	Zm00001eb181350_T001	411	46.27	8.56	64.6	89.93	−0.185	Nuclear
*ZmCHR14*	Zm00001eb186260_T001	1360	151.36	6.43	51.85	74.21	−0.675	Nuclear
*ZmCHR15*	Zm00001eb187690_T003	2018	231.23	5.38	50.32	77.33	−0.654	Nuclear
*ZmCHR16*	Zm00001eb220010_T002	879	99.64	8.65	48.91	81.95	−0.501	Nuclear
*ZmCHR17*	Zm00001eb229760_T001	2208	246.56	8.85	56.41	66.3	−0.815	Nuclear
*ZmCHR18*	Zm00001eb251100_T003	1975	226.47	5.29	52.17	76.95	−0.653	Nuclear
*ZmCHR19*	Zm00001eb255780_T002	968	107.38	6.42	49.17	81.27	−0.46	Nuclear
*ZmCHR20*	Zm00001eb280740_T001	3828	414.73	5.07	52.19	71.07	−0.647	Nuclear
*ZmCHR21*	Zm00001eb297280_T001	2036	225.90	5.87	44.01	97.49	−0.188	Nuclear
*ZmCHR22*	Zm00001eb322510_T001	850	95.87	8.63	51.51	76.42	−0.48	Nuclear
*ZmCHR23*	Zm00001eb326710_T001	1202	135.36	8.39	50.3	88.78	−0.38	Nuclear
*ZmCHR24*	Zm00001eb328680_T001	717	78.84	9.66	50.48	94.3	−0.153	Mitochondrial
*ZmCHR25*	Zm00001eb329060_T003	854	95.16	8.68	41.69	90.91	−0.265	Nuclear
*ZmCHR26*	Zm00001eb330720_T003	1709	193.32	6.68	48.47	84.96	−0.375	Nuclear
*ZmCHR27*	Zm00001eb331510_T001	1214	137.93	8.05	43.49	82.06	−0.404	Nuclear
*ZmCHR28*	Zm00001eb341650_T002	1102	127.34	5.25	48.09	72.99	−0.853	Nuclear
*ZmCHR29*	Zm00001eb369880_T003	1239	138.32	5.15	52.28	72.32	−0.513	Nuclear
*ZmCHR30*	Zm00001eb379660_T002	2254	249.35	6.04	53.3	84.63	−0.444	Nuclear
*ZmCHR31*	Zm00001eb394610_T002	1380	157.94	8.79	46.64	82.78	−0.553	Cytoplasmic
*ZmCHR32*	Zm00001eb404510_T002	904	101.65	6.55	46.13	94.21	−0.214	Nuclear
*ZmCHR33*	Zm00001eb416780_T001	950	106.54	6.61	43.06	84.57	−0.442	Nuclear
*ZmCHR34*	Zm00001eb417900_T002	1110	126.98	6.04	52.86	81.82	−0.624	Nuclear
*ZmCHR35*	Zm00001eb431470_T001	979	109.84	6.29	51.09	78.77	−0.464	Nuclear
*ZmCHR1*	Zm00001eb017000_T001	1398	159.89	8.11	48.88	83.53	−0.529	Nuclear
*ZmCHR2*	Zm00001eb046650_T001	1481	168.18	5.46	51.9	76.91	−0.674	Nuclear
*ZmCHR3*	Zm00001eb055640_T002	837	94.16	5.46	45.07	92.19	−0.417	Nuclear
*ZmCHR4*	Zm00001eb073870_T001	973	109.04	6.12	52.05	81.01	−0.525	Nuclear
*ZmCHR5*	Zm00001eb076670_T001	1048	116.95	5.85	42.11	83.11	−0.533	Nuclear

## Data Availability

All relevant data are provided as figures or tables within the paper.

## Supplementary Materials

The following supporting information can be downloaded at: https://www.mdpi.com/article/10.3390/ijms25179686/s1.
